# An Optimized DNN Model for Real-Time Inferencing on an Embedded Device

**DOI:** 10.3390/s23083992

**Published:** 2023-04-14

**Authors:** Jungme Park, Pawan Aryal, Sai Rithvick Mandumula, Ritwik Prasad Asolkar

**Affiliations:** College of Engineering, Kettering University, Flint, MI 48504, USA

**Keywords:** ADAS, object detection, convolution neural network, TensorRT, deep neural network, transfer learning, embedded devices

## Abstract

For many automotive functionalities in Advanced Driver Assist Systems (ADAS) and Autonomous Driving (AD), target objects are detected using state-of-the-art Deep Neural Network (DNN) technologies. However, the main challenge of recent DNN-based object detection is that it requires high computational costs. This requirement makes it challenging to deploy the DNN-based system on a vehicle for real-time inferencing. The low response time and high accuracy of automotive applications are critical factors when the system is deployed in real time. In this paper, the authors focus on deploying the computer-vision-based object detection system on the real-time service for automotive applications. First, five different vehicle detection systems are developed using transfer learning technology, which utilizes the pre-trained DNN model. The best performing DNN model showed improvements of 7.1% in Precision, 10.8% in Recall, and 8.93% in F1 score compared to the original YOLOv3 model. The developed DNN model was optimized by fusing layers horizontally and vertically to deploy it in the in-vehicle computing device. Finally, the optimized DNN model is deployed on the embedded in-vehicle computing device to run the program in real-time. Through optimization, the optimized DNN model can run 35.082 fps (frames per second) on the NVIDIA Jetson AGA, 19.385 times faster than the unoptimized DNN model. The experimental results demonstrate that the optimized transferred DNN model achieved higher accuracy and faster processing time for vehicle detection, which is vital for deploying the ADAS system.

## 1. Introduction

Perceiving the environment using sensors is essential to autonomous systems such as Autonomous Vehicles (AV), Advanced Driver Assist Systems (ADAS), robotics, drones, etc. Autonomous systems can identify their surroundings using various sensors and Artificial Intelligence (AI) technologies. The camera sensor is one of the most common sensors used for environmental perception because of its low cost and ability to identify different types of objects. During the last two decades, computer vision systems for environmental perception have achieved state-of-the-art performance due to the emerging Deep Neural Network (DNN) technologies. Since LeCun et al. [1] proposed a Convolutional Neural Network (CNN) for automated handwritten digit recognition in 1998, many deep CNN learning models [2,3,4,5,6,7,8] have been proposed. Recently, those CNN models based on DNN have achieved a remarkable object classification and detection breakthrough.

Low latency and high accuracy are required when DNN models are deployed for automotive applications because response time and reliability are critical. The main drawback of DNN-based detection systems is that they need high computational power, so deploying them on vehicles for real-time inferencing is challenging. A Graphics Processing Unit (GPU) is used to accelerate the processing of the DNN-based models to provide high computational power. GPU technologies are continually evolving and increasing in computing power. In addition, many edge computing platforms have been released starting in 2015. These edge computing devices have high costs and require high power consumption. Due to the high price, using them to deploy the DNN module massively will be challenging.

Recently, onboard computing units such as the NVIDIA Jetson series [9] (Nano, NX, AGX, etc.) have been introduced on the market. These onboard computing platforms are ideal for real-time AI system deployment because they can run the DNN models in parallel and become the ideal engine for Autonomous Driving (AD) cars and ADAS [10]. Previous research on deploying the DNN models for real-time inferencing [11,12,13,14] has involved either deploying on expensive and high-power consumption devices, lacking a quantitative assessment of the onboard computing platforms’ performance, or high latency issues. Since little research has been conducted on deploying the DNN models on these onboard computing units, studies on how to deploy the DNN model on them and evaluate their latency and performance are necessary.

**Figure 1:**
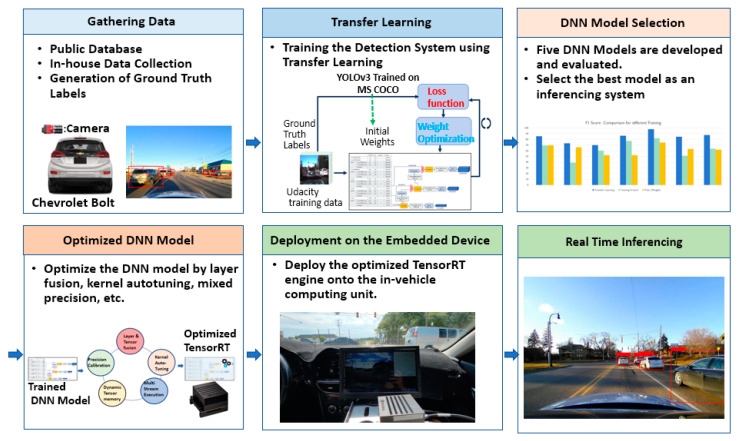
The overall architecture of the real-time inferencing system on the in-vehicle embedded device.

In this research, the authors focus on deploying the computer-vision-based vehicle detection system for real-time inference on the embedded device. The overall developing procedures for the proposed system are depicted in Figure 1. To develop the vehicle detection system, images from various scenarios were collected using a camera sensor mounted on a Chevy Bolt and labeled the ground truth locations of the target objects on the collected images. The available public data set was also appropriately processed. Five DNN models were retrained using the transfer learning technology to reuse the information from the pre-trained DNN, YOLOv3 [15]. The developed model was optimized and converted into a TensorRT to deploy the system for real-time inferencing [16]. The DNN model can be optimized by fusing layers and tensors and tuning kernels. Finally, the optimized DNN model was deployed on the embedded device in a vehicle and the performance was evaluated in terms of accuracy and speed using various testing scenarios.

In this paper, Section 2 presents a detailed literature review of object detection and deployment of the perception system. Next, Section 3 discusses and illustrates procedures for developing the optimized DNN model using transfer learning. The evaluations of the five different DNN models developed using the transfer learning technology are shown in Section 4. Finally, the future research scope and conclusion are discussed in Section 5. 

## 2. Related Work

Perception sensing in AD and ADAS involves collecting data from vehicle sensors and processing these data into an understanding of the environment around the vehicle. The camera is crucial in understanding the scene around the car, so vision-based object detection is a vital part of the perception system. Because of these reasons, object detection for ADAS has received great attention over the last decade. Many earlier object detection algorithms were built based on handcrafted features due to a need for more effective image representation [17,18]. The performances of these handcrafted features were saturated until the rebirth of Convolutional Neural networks (CNNs) in 2012. Since Alex Net [19] won the ImageNet Large Scale Visual Recognition Challenge (ILSVRC) [20] and achieved a significant milestone in the field of deep learning, a CNN renaissance has started [4,21,22].

Nowadays, the popular DNNs for object detection in images are categorized into two groups: two-stage detectors vs. one-stage detectors. The two-stage detectors are region-based proposal DNN models such as RCNN [4], Fast RCNN [23], and Faster RCNN [2]. To localize and segment objects, those models propose the regions and apply CNNs to classify the object types. For example, Faster R-CNN combines two CNNs, one that proposes regions and the other that uses those proposals to output detections [2]. When the image is fed through the Region Proposal Network (RPN), it outputs a set of rectangular object proposals using the sliding window approach. At each sliding window location, they simultaneously predict multiple region proposals relative to reference boxes known as anchors. The non-maximum suppression is adapted based on the threshold score to develop a single object proposal to reduce the redundancy with the object proposal. 

On the other hand, one-stage DNN detectors include You Only Look Once (YOLO) [5,15], Single Shot multi-box Detector (SSD) [24], and RetinaNet [25]. For example, in a unified object detection model, YOLO [5] takes a single input image, divides the image into S × S grid cells, and proposes bounding boxes and probabilities for each region. The model directly regresses from the input image to a tensor, representing class scores and locations of each object. The input images need to go through the network once, so the model processes images faster. 

Regarding deploying DNN models, Hui et al. [11] presented selection guidance and characterized a GPU platform based on accuracy, latency, and energy efficiency. They stated that edge AI processors could deliver better efficiency from the experimental observations. Tiny-YOLO and YOLOv2 models were deployed on three-edge AI platforms. The authors showed that the deployed systems had better latency and energy efficiency than the GPU platform. In [12], authors presented an integrated framework using an Nvidia Drive PX2 as an individual module that modified image streaming functionality to make the input image format compatible with the object detection framework to deploy DNN modules. They conducted experiments on the prototype vehicle and achieved 51 ms processing time on the Nvidia Drive PX2 platform. However, the Nvidia Drive PX2 platform is still expensive and requires high power consumption. 

Cantero et al. [13] discussed several quantization levels applied to a collection of deep learning models to accelerate deployment for real-time applications on resource-constrained devices, including Variscite i-MX8M-Plus board and Edge TPU (Tensor Processing Unit) Coral Dev Board. They reviewed significant challenges in developing embedded deep learning applications and carried out a benchmark of embedded hardware platforms. They could deploy “CenterNet” and “SSD” architectures on both hardware devices. However, they did not make a quantitative assessment of the performance of the converted models, which were only done by visual inspection of the detected object. In [14], the authors developed a method of region detection followed by target detection to detect small-scaled targets from large-scale remote sensing images with a light-weighted neural network of 17 million parameters. They used different scale information gathered by featured pyramid structure by integrating feature maps. However, the average inference time took 279 ms per network input on “MAXN” power modes, and there was a need to optimize the network to reduce the inferencing time. Stäcker et al. [26] discussed various approaches to optimize the run time performance of DNNs for object detection on edge AI devices. The techniques employed include model compression, quantization, and pruning which can reduce the size and complexity of the model and improve its runtime performance. They found that the available power supply in the embedded environment significantly impacted the runtime.

## 3. Development of the Obstacle Detection System Based on Transfer Learning

Developing object detection systems using DNNs involves many procedures, from data preparation for machine learning to deploying the system on the embedded device for real-time inferencing. An object detection system generally has two major phases: training and inferencing. Training is the phase in which the neural network algorithm tries to learn from the data. Each network layer is assigned random weights at the beginning of training. The training algorithm runs a feed-forward pass through the data and makes predictions. The predictions are then compared against the actual labels, and an error is computed via a loss function. This error is then backpropagated through the network, and weights in layers are updated accordingly via a weight update algorithm, such as the Gradient Descent algorithm [27]. After many iterations to update associated weights to reduce the loss function, the training will be stopped when the prediction error is less than the threshold. On the other hand, the inference phase is the stage in which a trained model is used for real-time predictions. It is a production phase in which the model is deployed on the embedded computer unit to generate the real-time object detection output from a live-streamed camera sensor.

Developing a robust vehicle detection system with a relatively small dataset is challenging. Additionally, collecting data and generating ground truth data is a tedious and time-consuming task. However, utilizing the pre-trained DNN models trained with a large amount of data, the already-learned features in the pre-trained DNN model can be generalized across most types of images. By transferring those learned features from the pre-trained DNN model to the new DNN model in a similar domain, we can develop a new DNN model with relatively small data samples. 

### 3.1. Pre-Trained Object Detection Model: YOLOv3

**Figure 2:**
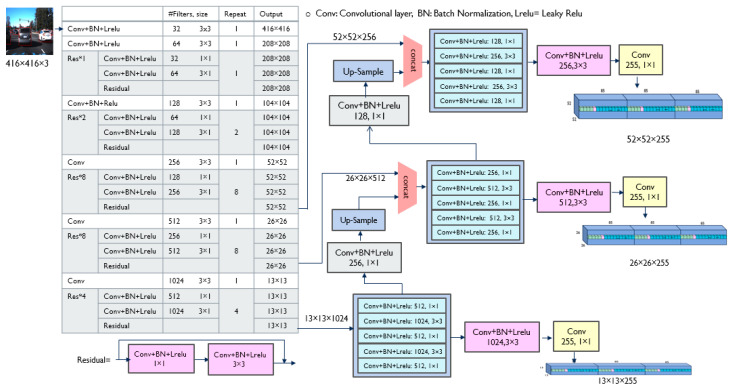
The architecture of YOLOv3. Note: # filters = the number of filters.

To develop a reliable vehicle detection system for collision avoidance, YOLOv3 [15] was chosen as the pre-trained network. YOLOv3 is one of the most popular DNN models, and has been trained on a large MS COCO (Microsoft Common Objects in Context) [28] dataset that included a 200 k labeled image as a dataset. YOLOv3 uses a trained DarkNet53 as feature extraction layers and conducts detections at three different scales, as shown in Figure 2. The residual blocks in the DarkNet53 are represented as Res*n in Figure 2, where n is the number of repetitions of the residual block. The residual layer in the residual block is based on deep residual learning by implementing feed-forward neural networks with shortcut connections (or skip connections) proposed by He et al. [3]. This allows neural networks to deepen without exhibiting a higher training error and to display higher accuracy with increasing depth compared to previously proposed networks. 

**Figure 3:**
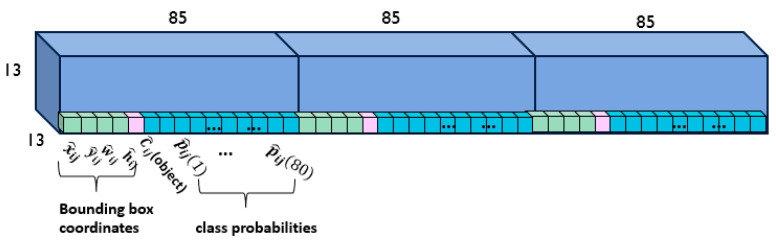
The 13 × 13 × (85 × 3) output layer in YOLOv3.

In each scale of YOLOv3, the input image is divided into S × S grids, and each grid is responsible for detecting the object whose center falls within that grid. Each grid predicts B bounding boxes (B = 3 in YOLOv3), and each bounding box requires information about the center of the bounding box (x, y), the width and height of the bounding box (w, h), and the confidence score. The confidence score of the box, C(object), represents the probability that the box contains the object. YOLOv3 generates K-class (K = 80) probability values for each bounding box in each grid cell. Figure 3 shows the predicted outputs at one scale, 13 × 13 grid cells. Each grid cell has three boxes, and each box in the grid cell contains 85 outputs, including the bounding box information, confidence score, and probabilities of 80 classes.

The loss function, *Loss*, used in the YOLOv3 model contains three loss functions: the localization loss for bounding box prediction, the loss from the box confidence score, and the classification loss for conditional class probabilities presented in Equations (1)–(4) [29]. In YOLOv3, the Binary Cross Entropy (BCE) function is used instead of the mean squared error function when calculating confidence loss and classification loss. The *BCE* function is defined in Equations (5) and (6).

(1) $$Loss=Loss_{localization}+Loss_{confidence}+Loss_{classification}$$

(2) $$Loss_{localization}=\lambda_{1}\sum_{i=0}^{S^{2}}\sum_{j=0}^{B}I_{ij}^{obj}\;\left[{\left(x_{ij}-{\hat{x}}_{ij}\right)}^{2}+{\left(y_{ij}-{\hat{y}}_{ij}\right)}^{2}\right]+\;\lambda_{1}\sum_{i=0}^{S^{2}}\sum_{j=0}^{B}I_{ij}^{obj}\;\left[{\left(w_{ij}-{\hat{w}}_{ij}\right)}^{2}+{\left(h_{ij}-{\hat{h}}_{ij}\right)}^{2}\right],$$

(3) $$Loss_{confidence}=\sum_{i=0}^{S^{2}}\sum_{j=0}^{B}I_{ij}^{obj}BCE(C_{ij,\;}\;{\hat{C}}_{ij})+\lambda_{2}\sum_{i=0}^{S^{2}}\sum_{j=0}^{B}I_{ij}^{noobj}\;BCE(C_{ij,\;}\;{\hat{C}}_{ij})(1-I_{ij}^{obj})$$

(4) $$Loss_{classification}=\overset{S^{2}}{\underset{i=0}{\sum }}I_{i}^{obj\;}\overset{B}{\underset{j=0}{\sum }}\underset{k\in classes}{\sum }BCE(p_{ij}(k),\;{\hat{p}}_{ij}(k)),$$

(5) $$BCE(C_{ij\;},\;{\hat{C}}_{ij})=-(\;C_{ij}log{\hat{C}}_{ij}+(1-C_{ij\;})\log(1-{\hat{C}}_{ij}))$$

(6) $$BCE(p_{ij}(k),\;{\hat{p}}_{ij}(k))=-(\;p_{ij}(k)log{\hat{p}}_{ij}(k)\;+(1-p_{ij}(k))\log(1-{\hat{p}}_{ij}(k))\;),$$

where $I_{ij}^{obj}$ = 1 if the *j*th bounding box in grid cell *i* is responsible for detecting the object, otherwise 0; $I_{ij}^{noobj}$ is the complement of $I_{ij}^{obj}$; $\lambda_{1}$ and $\lambda_{2}$ are the parameters to control the penalties from the localization and confidence loss, respectively; and ${\hat{p}}_{ij}(k)$ is the conditional probability for class *k* in the *j*th bounding box of grid cell *i.* The input’s final inferences are generated by applying the non-maximal suppression algorithm.

### 3.2. Transfer Learning for Obstacle Detection

To develop a reliable vehicle detection system, the pretrained DNN model YOLOv3 is utilized. Transfer learning is referred to as “Knowledge Transfer”, “Multi-task Transfer”, and “Knowledge Consolidation” in early studies [30,31]. Transfer learning reuses a pre-trained DNN model extensively trained on large datasets to solve a new task with a similar domain [4,17,21,22]. The typical training procedure for object detection includes taking a large set of data from different classes on which the inference is to be done. The training takes much time and computation power for such a large dataset. These pre-trained deep neural network models have learned primitive features in images such as object shapes, edges, and lighting with visual image data in its convolution layers using a huge amount of data samples. Because these features are generalized across most types of images, utilizing those learned features from big data in the existing DNN model for a new model with relatively small data samples provides better accuracy than training the new model from scratch [32].

**Figure 4:**
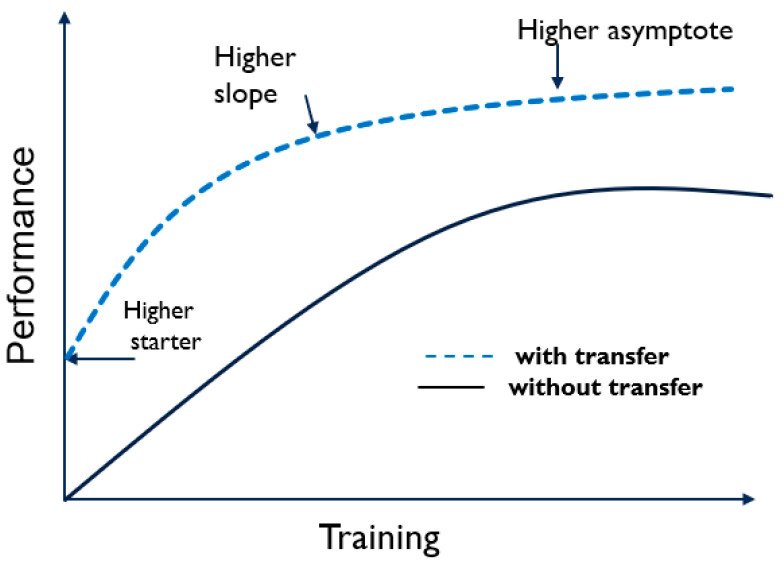
The benefits of transfer learning.

In addition, transfer learning reduces the time needed to train a network and removes the need for an extensive data set along with complexities that come with large data, such as labeling and checking the uniformity across the training class. Developing a DNN-based object detection system with small image data samples is challenging and performs poorly. However, by utilizing a pre-trained DNN model trained with big data, the learned primitive features can be transferred to a new system with a smaller image dataset. Transfer learning generally achieves a high start, higher slope, and asymptote, as presented in Figure 4. More details on the benefits looked for by transfer learning are discussed in [30]. 

**Figure 5:**
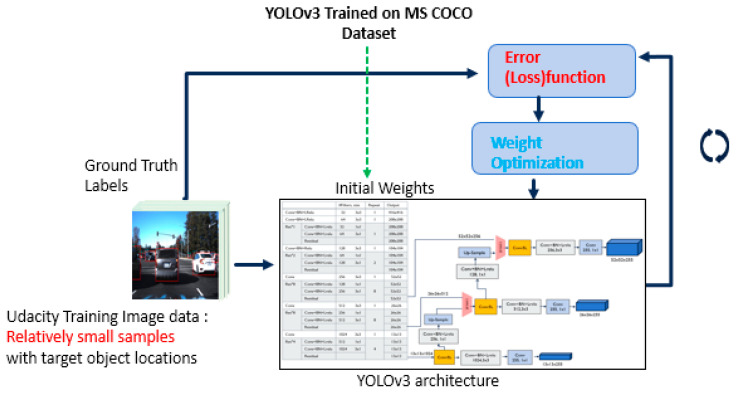
Transfer learning using the Udacity dataset.

The procedures for transfer learning are summarized in the following steps, as presented in Figure 5:

1. Unfreeze the several layers in the pre-trained DNN model while keeping all the other layers frozen;
2. The network runs through a feed-forward pass through the network using the training data samples for the new model;
3. The class scores predicted at the output layers are compared against the actual labels in the training dataset for transfer learning;
4. The error for the classification is then computed using the loss function;
5. The error is then backpropagated through the network while updating the weights using Stochastic Gradient Descent (SGD) for optimization.

Repeat Steps 1–5 until the loss function converges or a certain number of training epochs is completed. 

**Figure 6:**
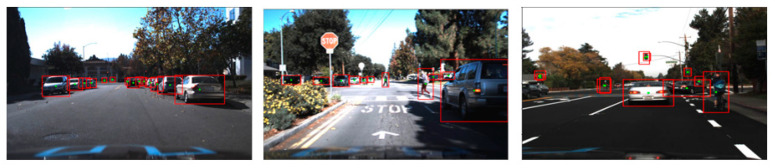
Training data sample images and visualized labels. The red boxes in the images are the ground truth locations of the target objects.

The Udacity dataset [33] was used to retrain the pre-trained YOLOv3 model for transfer learning. The Udacity dataset comprises 22,065 labeled images collected in various driving scenarios during daylight. The sample images of the training dataset are given in Figure 6. The dataset was split into 80:20 for training the new model and evaluating the trained model. A total of 17,652 images in the Udacity dataset were used to re-trained the DNN model to develop an accurate detection system using transfer learning. The pre-trained YOLOv3 model was developed using the MS COCO dataset [28] to classify 80 different categories of objects in images. The transfer learning converts the pre-trained YOLOv3 DNN model into a vehicle detection system that detects vehicles in images. Transfer learning reuses the generalized features (object’s shape, edges, corners, etc.) learned in YOLOv3 to develop a new vehicle detection model with relatively small data samples.

The model developed by transferring learning was deployed on the in-vehicle computing unit. The chosen embedded GPU device was the NVIDIA^®^ Jetson AGX Xavier™ Developer Kit. The NVIDIA Jetson AGX was selected because it is in a compact form factor (4.2 × 4.2 × 4 inches) with a reasonable price. In addition, this embedded system has a powerful computing power of up to 32 TOPs (Tera operations per second) with a 512-core Volta GPU. Furthermore, it accelerates AI applications efficiently and powerfully in an embedded module under 30 W because it is supported by CUDA^®^, cuDNN, and TensorRT™ software libraries to optimize the DNN modules [16]. However, the inferencing time using an un-optimized DNN model is not acceptable for real-time applications. That is why optimizing the DNN model is an indispensable step in running the DNN model for real-time applications.

### 3.3. Optimization of the DNN Model for Real-Time Inferencing 

**Figure 7:**
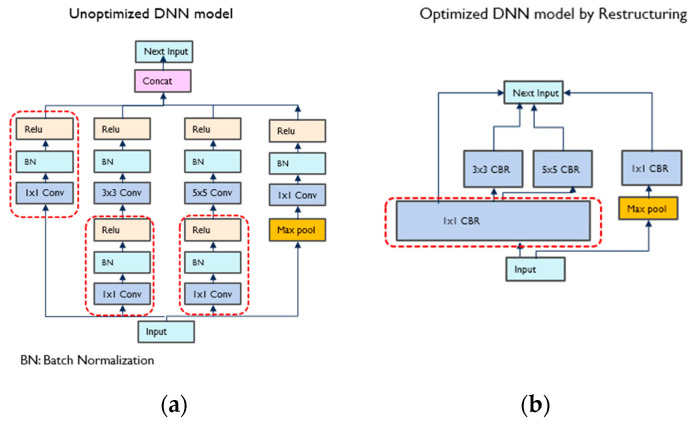
Network graph compression to optimize the DNN model: (**a**) the network graph before optimization; (**b**) the network graph after the model compression.

Optimizing the DNN system to make it run on the in-vehicle embedded device is an essential step. The DNN model optimization can be done in several ways. Figure 7 summarizes how TensorRT generates the optimized TensorRT engine by compressing the DNN model and hardware mapping [16]. During model compression, the optimization procedure restructures the network graph in three different ways in order to perform the operations more efficiently: (1) Kernels are vertically fused to perform the sequential operation together. For example, the three layers of convolution, batch normalization, and Relu in Figure 7a are merged into one layer, CBR in Figure 7b. (2) Layers are fused horizontally to a single wider kernel if they share the same input and filter size with different weights. For example, layers in red-dotted rectangles in Figure 7a are fused vertically first and then combined horizontally to generate a 1 × 1 CBR layer in the red-dotted box in Figure 7b. (3) To reduce the computation, the useless layers are eliminated by analyzing the model. The dead layers, which are required only in the training and validation of the model, are useless and eliminated by parsing the network model to reduce computation. Figure 7a is the network graph before the model compression, and Figure 7b shows the compressed network graph restructured. 

In addition, half-precision acceleration is conducted during the model compression to improve computing efficiency. Half-precision arithmetic operations require less memory and computing power and are much faster than single- or double-precision data. During the training of the DNN model, full 32-bit precision was used for the accuracy of gradient backpropagation [27]. However, half-precision FP16 or INT8 can be utilized for the inferencing task because it does not require backpropagation. Kernel auto-tuning optimizes software for highly efficient execution on a target hardware platform. In hardware mapping, kernel auto-tuning is performed by selecting the optimal pre-implemented algorithms and the optimal batch size based on the target GPU platform to maximize parallel operations. Furthermore, memory footprints are removed, and memory reuse is improved by designing streaming technology in CUDA to maximize parallel operations [16].

**Figure 8:**
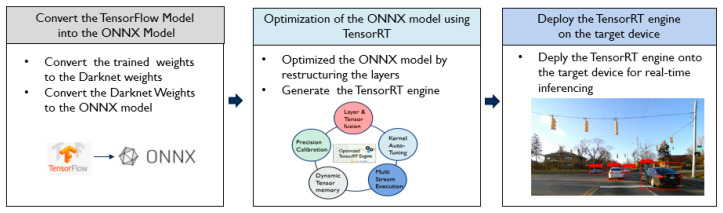
Optimization and deploy procedures.

The optimization procedures are summarized in Figure 8. First, the vehicle detection model is retrained on the TensorFlow-Keras framework using transfer learning. The TensorFlow-Keras model is converted into the Darknet weights for feasibility and easy conversion. Then, the Open Neural Network Exchange (ONNX) model [34] is generated from the Darknet weights. ONNX [34] converts the deep learning models from different frameworks to a common set of operators, which are common groups of building blocks of deep learning. Finally, the ONNX parser in TensorRT parses the ONNX model. Then, TensorRT optimizes the ONNX model on the target embedded device and generates the TensorRT engine. The TensorRT engine is able to run for real-time inferencing without the overhead of a framework.

## 4. Experiments on the Real-Time Inferencing System for Obstacle Detection

Five different DNN models were developed by freezing different layers using the transfer learning technology. Those five different DNN models are defined below:

- TL Model #1: the DNN model is re-trained from scratch by freezing the Darknet53 body (185 layers) and retraining the whole 67 layers after the Darknet;
- TL Model #2: the DNN model is developed by re-training the last 15 layers;
- TL Model #3: the DNN model is developed by re-training the last 12 layers;
- TL Model #4: the DNN model is developed by re-training the last 6 layers;
- TL Model #5: the DNN model is developed by re-training the last 3 layers.

**Figure 9:**
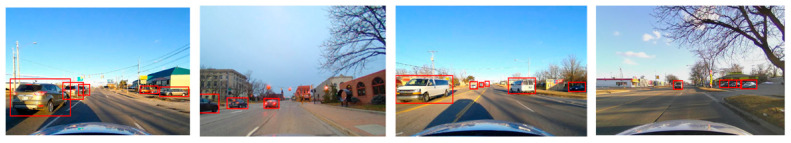
Sample images were collected and labeled to evaluate the DNN models. The red boxes in the images are the ground truth locations of the target objects.

The evaluations of the DNN models were conducted with the 8227 images, including 4364 Udacity testing samples separated from training and 3863 new images collected. The newly collected images shown in Figure 9 were labeled using MATLAB Image Labeler tool [35] for the ground truth locations of the target objects. The evaluations of the DNN models on the testing data samples were conducted on the laptop computer, Dell Alienware, with the processor 9th Gen Intel^®^ CoreTM i7. The performances of the DNN models for vehicle detection were measured with three metrics, Precision, Recall, and F1 score defined in Equations (7) and (8). F1 score is a harmonic mean of Precision and Recall takes both metrics into account. Instead of calculating the simple average of two metrics, Precision and Recall, the F1 score punishes the extreme values and balances both metrics.

(7) $$\mathrm{Precision}=\frac{\mathrm{TP}}{\left(\mathrm{TP}+\mathrm{FP}\right)}\;,\;\mathrm{Recall}=\frac{\mathrm{TP}}{\left(\mathrm{TP}+\mathrm{FN}\right)}\;,$$

(8) $$F1\;\mathrm{score}=2\times \frac{\mathrm{Precision}\;\times \mathrm{Recall}}{\left(\mathrm{Precision}+\mathrm{Recall}\right)}\;,$$

where TP = the total number of cases where the model detects positive samples correctly, FP = the total number of cases where the model incorrectly detects negative samples as positive samples, and FN = the total number of cases where the model misses the detection of positive samples.

**Figure 10:**
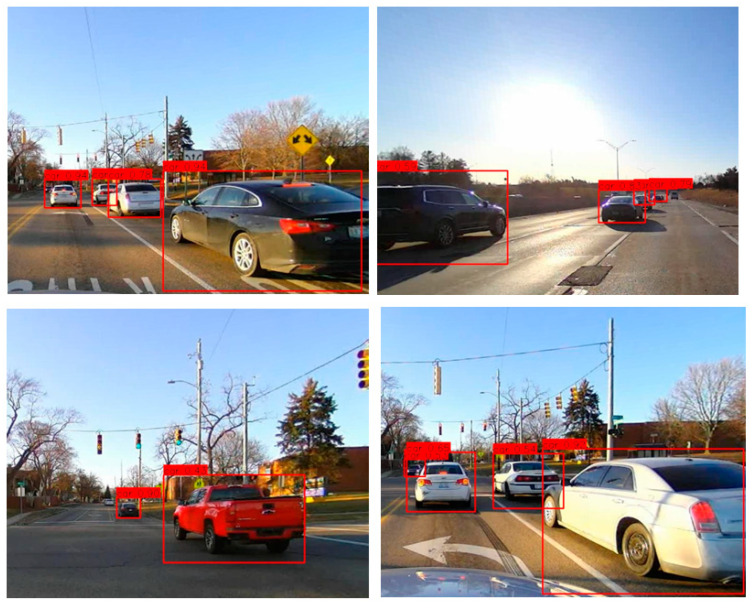
Examples of detection results by TL Model #4. The detected vehicles are marked with red bounding boxes.

Six different DNN models including the original YOLOv3 [15] were evaluated using the 8227 testing images. The evaluation results are summarized in Table 1. Considering Precision, Recall, and F1 score, the best performance model is TL Model #4, in which the last six layers are retrained using 80% of the Udacity data set. The three metrics, Precision, Recall, and F1 score of TL Model #4, are 90.09%, 93.07%, and 91.56%, respectively. Compared to the original YOLOv3 model, TL Model #4 improved by 7.1% in Precision, 10.8% in Recall, and 8.93% in F1 score. TL Model #4 reused most of the smaller details, such as edges and lines, in the pre-trained original YOLOv3 model, but weights on the final layers were fine-tuned to generalize the object classification. Figure 10 shows the inferencing outputs from the best performance model, TL Model #4.

On the other hand, TL Model #1, which was trained from scratch, shows poor performance because TL Model #1 did not utilize the primitive features learned in the original YOLOv3 model by training the whole 67 layers after Darknet53. The accuracy of the transfer learning model highly depends upon the similarity of the domains. It is also greatly affected by the quality and quantity of the data set used to retrain and the fine-tuning approach. The original YOLOv3 model contains the DarkNet53 as a feature extract network trained on the ImageNet dataset [20]. Additionally, the whole YOLOv3 model was re-trained with the MS COCO dataset, which is a comprehensive and diverse dataset. In TL Model #1, all the layers after the DarkNet53 were retrained on a relatively small Udacity dataset that was not necessarily as comprehensive as the MS COCO dataset. This explains why the F1 Score was lower than the original YOLOv3 model and signifies the importance of the fine-tuning approach while retraining the model using transfer learning. 

**Figure 11:**
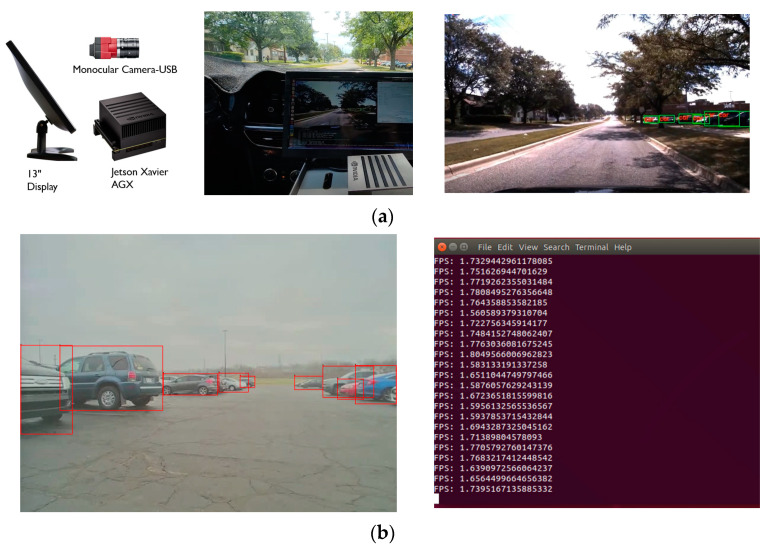
Deployment of the un-optimized transferred DNN model: (**a**) Hardware set-up for real-time inferencing; (**b**) the example output of the un-optimized transferred DNN model on Jetson AGX with fps information.

The best performing DNN model, TL Model #4, was deployed on the NVIDIA Jetson AGX Xavier directly, and the inferencing time was measured. In Figure 11a, one RGB camera is mounted on the windshield in the testing vehicle, a Chevy Bolt. One NVIDIA Jetson AGX and a 13-inch display device were placed near the dashboard to monitor the live streaming outcomes, as shown in Figure 11a. The sample detection result is presented in Figure 11b, where the red bounding boxes are the detected vehicles in the image. However, the average inferencing time of the DNN model, TL Model #4, on the Jetson AGX was 1.721 fps for the image frames with the size 640 × 480. It is not feasible to run the model for the real-time inferencing task on the embedded device. 

Since the low response time and high accuracy of automotive applications are critical factors when the system is deployed, the best model, TL Model #4, is optimized by following the procedures explained in Section 3.3. It is converted into the ONNX model first, and then the TensorRT engine:

1. Convert the trained model into the ONNX mode;
2. The ONNX model is converted into TensorRT engine. During the conversion, the network graph is restructured to perform the operations more efficiently.

**Figure 12:**
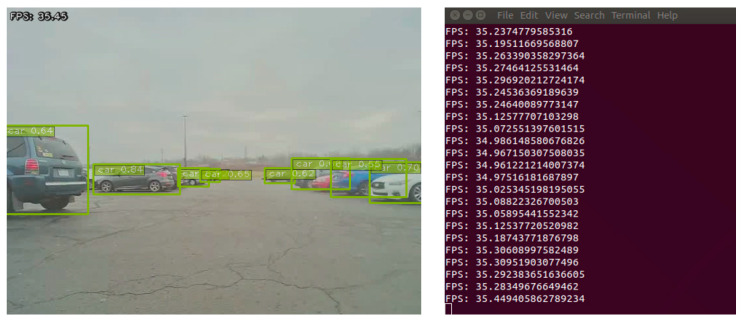
Example of detection results by the optimized DNN model, TL Model #4.

The TensorRT engine is deployed on the NVIDIA Jetson AGX device to run real-time inference. The optimized TL Model #4 runs on the embedded device with an average inferencing time of 35.082 fps for the image frames with the size 640 × 480. The optimized TL Model #4 can perform inference 19.385 times faster than the un-optimized TL Model #4. Figure 12 presents real-time inference with the optimized TL Model #4. 

## 5. Conclusions and Future Scope

This paper addresses deploying the DNN model on an embedded device for automotive applications. Low response time and high accuracy are critical factors for automotive applications when the system is deployed for real-time inferencing. The novelties of the proposed vehicle detection system are summarized as follows: Using the transfer learning technology, five different DNN models were developed and evaluated. The best performing transferred model, TL Model #4, achieved 90.09% in Precision, 93.07% in Recall, and 91.56% in F1 score. The transferred model, TL Model #4, improved by 7.1% in Precision, 10.8% in Recall, and 8.93% in F1-score compared to the original DNN model’s performance. The best performing model, TL Model #4, has been optimized to reduce the processing time using TensorRT. The optimization process restructures the model layers by fusing horizontally and vertically and accelerates half-precision FP16 or INT8 for inferencing to improve computing efficiency. Finally, the optimized TL Model #4 was deployed on the NVIDIA embedded in-vehicle computing device to run the program in real-time. The average processing time for 640 × 480 image frames was 35.082 fps on the NVIDIA Jetson AGX, which improves the processing time to 19.385 times faster than the original un-optimized TL Model #4. The experimental results demonstrate that the optimized transferred model, TL Model #4, achieved robust object detection accuracy and fast processing time, which is vital for deploying the ADAS system.

Based on the research outcomes of this paper, we have concluded as follows: (1) transfer learning is a way to develop an object detection system with relatively small data by utilizing the pre-trained DNN models. The model’s performance will differ based on the number of re-trained layers. If the size of data samples for transfer learning is relatively small, re-training the last few layers performed better than re-training the many layers from scratch. (2) The optimization process is essential to deploy the DNN model for real-time inferencing. In this research, the optimized DNN model can run 19.385 times faster than the un-optimized DNN model. The fast inferencing time makes the DNN model able to be deployed on a small-scale in-vehicle computing platform like NVIDIA Jetson AGX.

For future research, comparison experiments could be done on the various in-vehicle embedded devices for processing time. It is also essential to research how much the obstacle detection system can be improved by fusing different sensor information. In addition, further research on how to deploy a sensor-fused obstacle detection system for real-world implementation is required.

## Figures and Tables

**Table 1:** Evaluation results of the DNN models for vehicle detection.

DNN Models	Re-Trained Layers	Metrics		
		Precision × 100	Recall × 100	F1 score × 100
Original YOLOv3:	--	82.99	82.27	82.63
TL Model #1	67 layers	79.49	82.29	80.86
TL Model #2	15 layers	79.24	89.63	84.11
TL Model #3	12 layers	81.12	89.19	84.96
TL Model #4	6 layers	90.09	93.07	91.56
TL Model #5	3 layers	88.10	91.82	89.93

## Data Availability

The data presented in this study are available on request from the corresponding author.
